# Mutational status may supersede tumor size in predicting the presence of aggressive pathologic features in well differentiated thyroid cancer

**DOI:** 10.1186/s40463-022-00559-9

**Published:** 2022-03-04

**Authors:** Koorosh Semsar-Kazerooni, Grégoire B. Morand, Alexandra E. Payne, Sabrina D. da Silva, Véronique-Isabelle Forest, Michael P. Hier, Marc P. Pusztaszeri, Michael Tamilia, Richard J. Payne

**Affiliations:** 1 Faculty of Medicine, McGill University, Montréal, QC Canada; 2 Department of Otolaryngology-Head and Neck Surgery, Jewish General Hospital, McGill University, 3755 Côte-Sainte-Catherine Road, Montréal, QC H3T 1E2 Canada; 3 Department of Pathology, Jewish General Hospital, McGill University, Montréal, QC Canada; 4 Division of Endocrinology and Metabolism, Jewish General Hospital, McGill University, Montréal, QC Canada; 5 Marianopolis College – Health Sciences, Montréal, QC Canada

**Keywords:** Thyroid nodule, Proto-oncogene proteins p21(ras), Thyroid neoplasms, Ultrasonography, Mutation, Molecular diagnostic techniques

## Abstract

**Background:**

In clinical practice, thyroid tumor size plays a critical role in the staging of thyroid malignancies and in the selection of nodules that should undergo ultrasound-guided fine-needle aspiration. Thyroid tumor size is influenced by the elapsed time since the beginning of oncogenesis and by the presence of somatic mutations driving growth, such as *BRAF*^*V600E*^ mutations, associated with aggressive phenotypes, and *RAS-*like mutations, associated with more indolent behavior. Although large nodules are often considered to be more alarming, the true impact of tumor size on prognosis remains controversial. The aim of this study was to assess the relationship between mutational status, tumor size and aggressiveness, with emphasis on *BRAF*^*V600E*^ and *RAS*-like mutations.

**Method:**

We conducted a multicentric retrospective chart review in Montréal, Canada, of all patients who underwent thyroid surgery between January 2016 and December 2020, with well-differentiated thyroid cancer on final pathology, and who had undergone molecular testing revealing the presence of *BRAF*^*V600E*^ mutations or *RAS*-like mutations (*NRAS*, *HRAS* or *KRAS*).

**Results:**

We included 214 cases. There were 117 (54.7%) cases of *BRAF*^*V600E*^ and 97 (45.3%) cases of *RAS*-like mutations. The *BRAF*^*V600E*^ group was statistically associated with a smaller mean tumor size when compared with the *RAS* group of 1.55 cm and 2.04 cm, respectively. In a multivariate model, tumors with *BRAF*^*V600E*^ mutations were also more likely to display aggressive pathological features, including extra-thyroidal extension, lymph node metastasis, columnar cell features, tall cell histology, or hobnail histology (OR 26.69; 95% CI 11.15–70.81). In contrast, tumor size was not associated with pathologic aggressive features on multivariate analysis (OR 0.81; 95% CI 0.54–1.22).

**Conclusion:**

This study demonstrates that thyroid tumors expressing *BRAF*^*V600E*^ mutations correlate with aggressive pathologic features more than tumors expressing *RAS*-like mutations. When comparing tumors with *BRAF*^*V600E*^ and *RAS*-like mutations, the former were found to be smaller. As a result of this finding, this study suggests that molecular alterations may better predict aggressive pathologic features than the size of the tumor.

**Graphical abstract:**

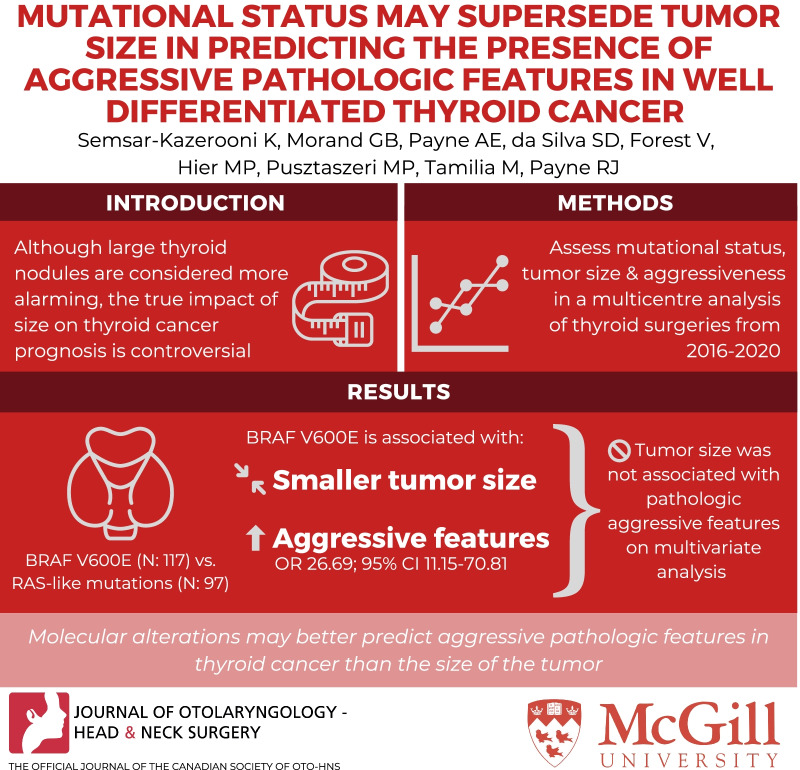

**Supplementary Information:**

The online version contains supplementary material available at 10.1186/s40463-022-00559-9.

## Introduction

Thyroid cancer is the most prevalent endocrine neoplasm worldwide [1], with papillary thyroid carcinoma being the most common subtype [2]. Thyroid ultrasound is commonly used as an initial diagnostic tool for thyroid nodules. Different sonographic features such as shape, echogenicity, margins, the presence of microcalcifications and size are used in many risk-stratification systems [3]. The Thyroid Imaging Reporting and Data System (TI-RADS) risk stratification tool recommends performing an ultrasound-guided fine-needle aspiration (USFNA) on mildly (TR3), moderately (TR4) and highly (TR5) suspicious nodules that are respectively larger than 2.5 cm, 1.5 cm, and 1 cm [4]. The Bethesda classification system is subsequently used to classify the cytology of the biopsied nodules according to their risk of malignancy [5].

After USFNA of thyroid nodules, cytologic diagnosis remains indeterminate (Bethesda Category III, IV, and V) in about 20–25% of cases [6–9]. For this reason, molecular testing has received extensive attention for its ability to improve risk stratification of indeterminate thyroid nodules [10], and avoid unnecessary diagnostic surgery [9]. Two of the most important and common mutations found in thyroid cancer are *BRAF*^*V600E*^ mutations and *RAS*-like mutations, which involve a family of three highly homologous isoforms (*NRAS*, *KRAS* and *HRAS*). The *BRAF*^*V600E*^ mutation is associated with more aggressive tumor behavior with papillary architecture, tall cell features, invasiveness, and frequent nodal metastasis [11–14]. On the other hand, *RAS*-like mutations are more commonly associated with indolent behavior, follicular growth, encapsulation, and a lower incidence of nodal metastasis [6].

In clinical practice, thyroid tumor size has been considered an important prognostic marker and plays a critical role in the selection of nodules that should undergo USFNA and the staging of thyroid malignancies [9, 15]. Although large nodules are often considered more alarming, the true impact of tumor size on prognosis remains controversial. Current data on the association between tumor size and prognosis is heterogeneous [16–21]. The impact of *BRAF*^*V600E*^ and *RAS*-like mutations on tumor size is also controversial, with some studies reporting smaller tumors when *RAS* mutations are present, and others reporting the contrary [22].

The aim of this study was to determine the impact of mutational status, specifically *BRAF*^*V600E*^ and *RAS*-like mutations, on tumor size. The second aim of this study was to compare the association between mutational status, size and pathological aggressiveness.

## Materials and methods

### Study design

A retrospective chart review was performed at the Jewish General Hospital (JGH) and McGill University Health Center (MUHC) in Montréal, Québec, Canada. Patients’ sociodemographic characteristics and oncologic characteristics (pre-operative cytology results, molecular testing results and final pathology results) were recorded. Ethics approval was obtained from both the McGill University Health Centre and CIUSSS West-Central Research Ethics Board in Montréal, QC, Canada (MP-37-2021-7517).

### Patient selection

Patients were included in the study if they had undergone thyroid surgery at the JGH or MUHC between January 2016 and December 2020, had a confirmed diagnosis of well-differentiated thyroid malignancy on final pathology, and had undergone molecular testing revealing the presence of *BRAF*^*V600E*^ mutations or *RAS-*like mutations (*NRAS*, *HRAS* or *KRAS*). Overall, 3 patients underwent Afirma™ testing, 157 underwent ThyGenext™ testing and 54 underwent ThyroseqV3™ testing. All these tests were shown to have equivalent diagnostic accuracies in previous studies [23]. Patients with other molecular mutations or with ≥ 2 concomitant mutations identified on molecular testing were excluded. The flow diagram of the patient selection process is provided in Additional file 1. Data collection results are provided in Additional file 2.

### Sample collection

After obtaining informed consent, specimen collection for molecular testing was performed with USFNA. The specimen was handled as per the company’s specific requirements and then sent to the appropriate lab for analysis.

### Pathology

Tumors were considered to be aggressive if they exhibited at least one of the following features on final postoperative pathology: extra-thyroidal extension (ETE), lymph node metastasis (LN+), or worrisome features/variants on pathology (columnar cell, tall cell or hobnail). All positive lymph nodes were incidental nodes seen as part of a prophylactic dissection, and micrometastases were excluded. An experienced thyroid pathologist reported on the final surgical specimens, highlighting the presence of any of the aforementioned features. Thyroid nodules were considered to exhibit tall cell or hobnail histology when > 30% of the tumor cells demonstrated tall cell or hobnail features. This cut-off was chosen based on the morphologic criteria reported in the 2017 WHO Classification of Tumors of Endocrine Organs [24, 25].

### Data analysis

The data was stratified into two groups according to the results of molecular testing (*BRAF*^*V600E*^ mutations group and *RAS* mutations group). Univariate analysis (Chi-square’s test) was used to compare Bethesda score distribution among the two groups. A threshold of *P* < 0.05 was determined for statistical significance. Simple logistic regression was performed to study the association of variables such as age, sex, McGill Thyroid Nodule Score (MTNS), pathological size and mutational status with aggressive features. The MTNS is a combined scoring system, used as a predictor for thyroid carcinoma, given clinical, radiological, and pathological findings of a certain nodule [26]. Subsequently, with the help of a multivariate model, we determined the association between aggressive pathological features and the following variables: age, sex, McGill Thyroid Nodule Score (MTNS), pathological size and mutational status. The fit and accuracy of the multivariate model was examined using the Hosmer–Lemeshow test. The association between pathological size and mutational status was assessed with a simple logistic analysis. All tests were performed using R software (R Foundation for Statistical Computing, Vienna, Austria).

## Results

A total of 1651 thyroid surgeries were performed in the targeted timeframe. Among them, 344 underwent molecular testing prior to their surgery and obtained positive results for the presence of suspicious molecular mutations. There were 130 cases excluded due to inconclusive final pathology (benign lesions or non-invasive follicular thyroid neoplasm with papillary like nuclear features) or due to the absence of isolated *BRAF*^*V600E*^ or *RAS-*like mutations on molecular testing. In total, 214 cases with either *BRAF*^*V600E*^ mutations or *RAS-*like mutations on pre-operative molecular testing were included in the study (Additional file 1: Fig. S1).

### Baseline characteristics

All the clinicopathological information available on medical records were analyzed. Baseline information including gender, age and the Bethesda score reflecting the USFNA biopsy results were collected for the 214 patients included in this study. There were 117 (54.7%) patients with *BRAF*^*V600E*^ mutation and 97 (45.3%) patients with *RAS*-like mutations (including *KRAS*, *NRAS* and *HRAS*). The mean age in the *BRAF*^*V600E*^ group was 45.7 years (SD 13.5) and 49.52 years (SD 13.6) in the *RAS* group. 22.2% of patients in the *BRAF*^*V600E*^ group were male compared to 25.0% in the *RAS* group. The distribution of Bethesda scores among both groups was statistically different. The majority of tumors with a *BRAF*^*V600E*^ mutation had higher Bethesda categories (5.1% category III, 0.9% category IV, 19.7% category V and 74.4% category VI), whereas most tumors harboring *RAS* mutations had lower Bethesda scores (34.0% category III, 39.2% category IV, 17.5% category V and 7.2% category VI; *P* < 0.0001). In the *BRAF* group all tumors were papillary thyroid cancers (PTC). In the *RAS* group, 95.9% of the cases were PTCs (93/97), 2.1% were follicular cell carcinomas (2/97) and 2.1% were Hurthle cell carcinomas (2/97) (Table 1).

**Table 1:** Aggressive features, relative risk of aggressiveness and pathological size in *BRAF*^*V600E*^ and *RAS*-like mutations

	BRAF^*V600E*^ (n = 117)	RAS (n = 97)
Age in years (SD)	45.7 (13.5)	49.6 (13.6)
Male, *N* (%)	26 (22.2)	24 (24.7)
Bethesda score distribution, *N* (%)		
III	6 (5.1)	33 (34.0)
IV	1 (0.9)	38 (39.2)
V	23 (19.7)	17 (17.5)
VI	87 (74.4)	7 (7.2)
Histological subtypes, *N* (%)		
Papillary carcinoma	117 (100)	93 (95.9)
Follicular carcinoma	0	2 (2.1)
Hurthle cell carcinoma Hurthle cell carcinoma	0	2 (2.1)
Any aggressive features, *N* (%)	89 (76.1)	9 (9.3)
Aggressive features, *N* (%)		
ETE*	32 (27.4)	1 (1.0)
LN+*	66 (56.4)	8 (8.2)
Tall cell	41 (35.0)	0 0
Columnar	2 (1.7)	0
Hobnail	10 (8.5)	0
Number of co-existing aggressive features in a single tumor, *N* (%)		
None	28 (23.9)	88 (90.7)
1 Feature	44 (37.6)	9 (9.3)
2 Features	32 (27.4)	0
3 Features	10 (8.5) 10 (8.5)	0
4 Features	2 (1.7)	0
5 Features	1 (0.9)	0
Mean pathological size in cm (SD)	1.55 (0.84)	2.04 (0.31)
Pathological size 0–1.00 cm, *N* (%)	37 (31.7)	18 (18.6)
Pathological size 1.01–1.50 cm, *N* (%)	33 (28.2)	22 (22.7)
Pathological size 1.51–2.00 cm, *N* (%)	19 (16.2)	17 (17.5)
Pathological size > 2.01 cm, *N* (%)	28 (23.9)	40 (41.2)

^*^ETE: Extra-thyroidal extension

^*^LN+: Lymph node metastasis

### Aggressive features and pathological size

**Fig. 1:**
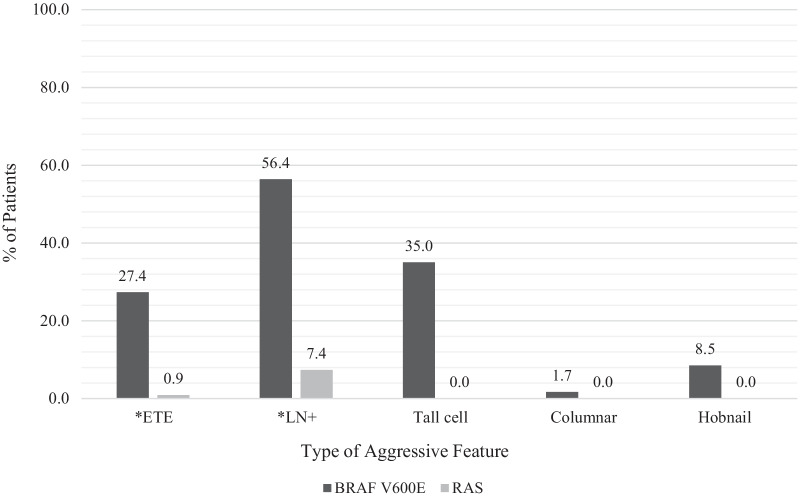
Prevalence of aggressive features in the *BRAF*^*V600E*^ group and in the *RAS* group. *ETE: Extra-thyroidal extension. *LN + : Lymph node metastasis

**Fig. 2:**
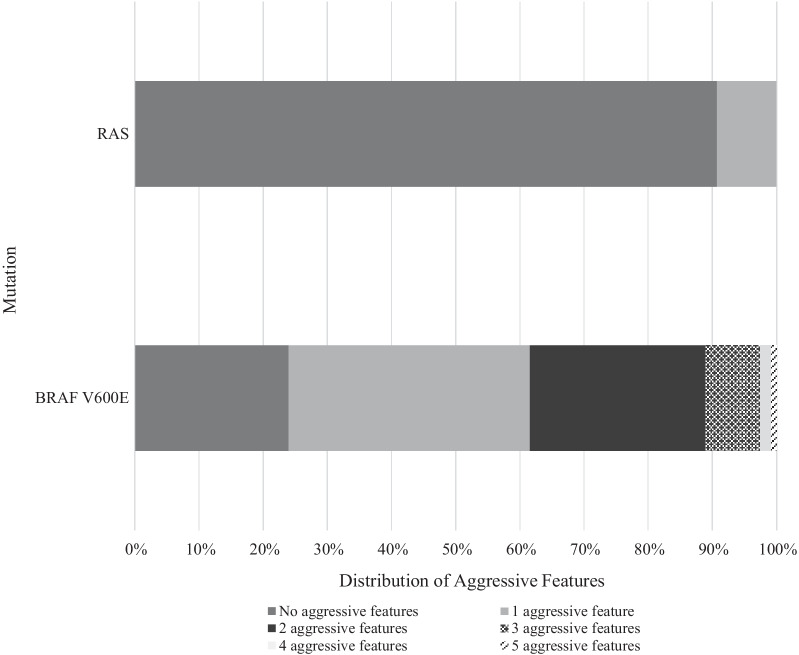
Number of aggressive features distribution in the *BRAF*^*V600E*^ group and in the *RAS* group

In patients with *BRAF*^*V600E*^ mutations, 76.1% (89/117) had at least one aggressive feature on final pathology report. 56.4% (66/117) of tumors in the *BRAF*^*V600E*^ mutation group had lymph node metastasis, 27.4% (32/117) had extra-thyroidal extension, 35.0% (41/117) were tall cell variants, 1.7% (2/117) exhibited columnar histology, and 8.5% (10/117) exhibited hobnail histology. In contrast, only 9.3% (9/97) of patients in *RAS*-like mutation group had aggressive features: lymph node metastasis (8.2%, 8/97) and extra-thyroidal extension (1.0%, 1/97). Importantly, no patients with *RAS*-like mutation were tall cell, columnar or hobnail variants (Table 1 and Fig. 1). The number of aggressive features in each tumor was also compared. 45/117 (38.5%) of tumors with *BRAF*^*V600E*^ mutation had more than one aggressive feature. In comparison, in the *RAS* group, all aggressive tumors had only one aggressive feature (Table 1 and Fig. 2). Overall, *BRAF*^*V600E*^ tumors were found to have qualitatively and quantitatively more aggressive features than *RAS*-like tumors. The *BRAF*^*V600E*^ mutation group was significantly associated with smaller tumors. The *BRAF*^*V600E*^ group was associated with a mean tumor size of 1.55 cm (SD 0.84), compared to the *RAS* group that was associated with a mean tumor size of 2.04 cm (SD 0.31) (Table 1).

### Logistic regression (aggressiveness)

On simple logistic regression, the presence of *BRAF*^*V600E*^ mutation (OR 31.08, 95% CI 14.51–73.78) and MTNS (OR 1.24, 95% CI 1.14–1.36) were associated with pathological aggressiveness. However, pathological size (OR 0.81, 95% CI 0.61–1.08), age (OR 0.98, 95%CI 0.96–1.00) and gender (OR 0.82, 95% CI 0.43–1.55) were not significantly associated with pathological aggressiveness. On multivariate analysis, mutational status was once again a significant predictor of aggressive pathological features (OR 26.69, 95% CI 11.15–70.81). In contrast, the association between pathological size and tumor aggressiveness was statistically insignificant (OR 1.00, 95% CI 0.68–1.49) (Table 2).

**Table 2:** Simple and multivariate analysis of association between covariates and aggressiveness

	Univariate analysis	Multivariate analysis	Hosmer–Lemeshow goodness of fit
	OR (95% CI)	OR (95% CI)	*P-*value
Mutational status (*BRAF*^*V600E*^)	31.08 (14.51–73.78)	26.69 (11.15–70.81)	0.222
Pathological size	0.81 (0.61–1.08)	1.00 (0.68–1.49)	
Age (years)	0.98 (0.96–1.00)	0.98 (0.96–1.01)	
Gender (Male)	0.82 (0.43–1.55)	0.84 (0.36–2.01)	
MTNS^*^	1.24 (1.14–1.36)	0.99 (0.90–1.09)	

^*^MTNS: McGill Thyroid Nodule Score

### Logistic regression (pathological size)

The association between mutational status and pathological size was assessed via two different methods: 1-A simple logistic regression with pathological size as an independent continuous variable, and in a simple logistic regression model with pathological size as an independent categorical variable (separated in 4 subgroups: 0–1.00 cm, 1.01–1.50 cm, 1.51–2.00 cm and > 2.01 cm. In the first method, for every additional 1.00 cm increase in size, the odds of presence of a *BRAF*^*V600E*^ mutation decreased by 0.61 (95% CI 0.45–0.82). In the second method, we can see an increase in the OR for decreasing size categories (Table 3).

**Table 3:** Simple logistic regression analysis of association between pathological size and presence of *BRAF*^*V600E*^ mutation

	OR (95% CI)
Pathological size (continuous), cm	0.61 (0.45–0.82)
Pathological size 0–1.00 cm, *N* (%)	2.03 (1.08–3.93)
Pathological size 1.01–1.50 cm, *N* (%)	1.34 (0.72–2.52)
Pathological size 1.51–2.00 cm, *N* (%)	0.91 (0.44–1.88)
Pathological size > 2.01 cm, *N* (%)	0.45 (0.25–0.80)

## Discussion

Substantial developments in clinical translational research have occurred in the past 5 years involving thyroid cancer. Molecular markers, such as *BRAF*^*V600E*^ and *RAS* mutations are outstanding examples in how a prognostic genetic marker can improve risk stratification and hence tailored management of patients with thyroid cancer, including those with conventionally low risks. The primary aim of this study was to determine the impact of mutational status, specifically *BRAF*^*V600E*^ and *RAS*-like mutations, on tumor size. It was found that *BRAF*^*V600E*^*-*positive tumors were smaller in size, when compared with *RAS*-like positive tumors. The second aim of this study was to compare the association between mutational status, size and pathological aggressiveness. *BRAF*^*V600E*^*-*positive tumors were more often presenting with aggressive features, such as nodal disease, extra-thyroidal extension and aggressive histological features/variants than *RAS*-like positive tumors. Although 0.49 cm is a clinically modest size difference, the multivariate analysis clearly demonstrated the strong correlation of mutational status with pathological aggressiveness, when compared to tumor size. In this model, the elevated odds ratio (26.69) and the wide confidence interval (11.15–70.81) could be explained by the wide gene expression variation noted across tumors with *BRAF*^*V600E*^-like mutations, in the literature. In a study published by The Cancer Genome Atlas Research Network (TGCA) in 2014, when comparing the *BRAF*^*V600E*^-like group with the *RAS*-like group, the former was overall predominantly less-differentiated, however their data also indicated that *BRAF*^*V600E*^-like tumors represent a diverse group with at least four major molecular subtypes with variable degrees of histological differentiation [27]. Therefore, despite the overall tendencies of *BRAF*^*V600E*^-like tumors to display more aggressive features, the wide diversity of genomic expression may account for the range of differences observed in our statistical analysis and the uncertainty regarding the prognostic and predictive power of *BRAF*^*V600E*^ mutations in the literature [28].

Previous studies examining the impact of *BRAF*^*V600E*^ and *RAS*-like mutations on thyroid cancer size have shown inconsistent results. Similarly, to the present study, a study by Kakarmath et al*.* reported a smaller average size of *BRAF*^*V600E*^*-*positive tumors when compared with tumors exhibiting *RAS*-like mutations, of 1.8 cm and size 2.5 cm, respectively [29, 30]. On the other hand, Al-Salam et al*.* found that thyroid tumors exhibiting *BRAF*^*V600E*^ mutations were associated with an increased size in a cohort of 90 adult patients in the United Arab Emirates [22]. A meta-analysis from Lee et al*.* found that the mean size of tumors exhibiting *BRAF* mutations ranged from 2.3 to 2.9 cm, whereas, in the absence of *BRAF* mutations, the mean size of tumors ranged from 1.8 to 2.7 cm [31]. Other studies were not able to find a statistically significant difference in size between *BRAF*^*V600E*^-associated tumors and *RAS*-associated tumors [9, 32, 33]. These discrepancies in the literature may be due to the differences in availability of high-resolution ultrasonography in different centers around the world. For instance, Al-Salam et al*.* reported that *BRAF*^*V600E*^ positive thyroid tumors were associated with a larger tumor size in a cohort from the United Arab Emirates [22]. This was not reflected in the context of the present study, which was conducted in Canada, a country with a publicly funded healthcare system, where patients with thyroid nodules have access to ultrasound without the need to pay. As a result, thyroid nodules may be picked at smaller sizes in a public healthcare system when compared to systems with financial barriers to medical imaging.

Primary tumor diameter has been described as a predictor of poor oncological outcomes in differentiated thyroid cancer [34]. Previous research has shown that larger thyroid tumor size is associated with an increased risk of recurrence, ETE, bilaterality, vascular invasion, lymph node metastases and distant metastases [35, 36]. For this reason, tumor size has traditionally been a key element in the staging, prognosis and management of thyroid tumors, with larger tumors often being considered more alarming than smaller ones. Moreover, thyroid tumor size plays a critical role in the selection of nodules that should undergo ultrasound-guided fine-needle aspiration, as suggested by many professional guidelines and risk stratification systems [37, 38]. Furthermore, treatment planning, including choice of surveillance and the appropriate extent of thyroid surgery (i.e., hemithyroidectomy versus total thyroidectomy and prophylactic central neck dissection) and staging of thyroid cancer often heavily rely on tumor size [37]. However, over the past few years, multiple major studies have suggested a progressive shift towards a new pathological classification of thyroid lesions, accounting for genotypic differences and consideration of individualized management depending on mutational profile [39–41]. Our findings indeed support this point of view, in regard to the current role and interpretation of tumor size in the management of thyroid nodules.

These results may also reflect the radiological and histological features of tumors with these mutations. Indeed, *BRAF*^*V600E*^-positive tumors are more commonly associated with suspicious ultrasound findings and lymph node involvement than tumors involving *RAS*-like mutations [33, 42]. These suspicious ultrasound findings may prompt earlier USFNA and earlier diagnosis of thyroid cancer. Moreover, it is also known that *BRAF*^*V600E*^-positive tumors are more likely to present with a higher Bethesda category on fine needle aspirate cytology, while tumors with *RAS*-like mutations are more commonly associated with lower Bethesda category [10]. Therefore, immediate surgical intervention is more likely to happen in a *BRAF*^*V600E*^-positive tumor, whereas tumors with *RAS* mutations are more likely to be managed with surveillance, with surgical removal only occurring in the presence of significant growth.

The results of the present study highlight the important role of molecular testing on predicting pathological aggressiveness, as smaller tumors may very well be more aggressive. These findings may be explained by the Knudson “two-hit hypothesis” [43]. While *RAS*-like mutations may stimulate clonal growth of follicular thyroid cells, this isolated mutation may be insufficient in itself to bring about the changes required for aggressive tumor behavior. In this case, a second molecular ‘’hit’’, including *TERT* or *p53* mutation, may be required for *RAS*-positive nodules to acquire these features [29]. Isolated *BRAF*^*V600E*^ mutations, on the other hand, may be sufficient to lead to aggressive tumor behavior by themselves. In this study, we excluded thyroid nodules exhibiting > 1 mutation from this study so that the effect of each mutation alone could be better compared.

There are several limitations in this study that should be recognized. First, the age and gender were the only demographic information collected in our database, which limits the accuracy of the multivariate model. Next, this paper was a cross-sectional study that only allowed us to evaluate aggressivity in terms of pathological features. Therefore, further longitudinal studies would be required to assess long-term oncological behavior associated with each of these mutations. Song et al. [44] and Yip et al. [41] displayed, respectively, higher recurrence rates and higher 5-year risk of distant metastasis with isolated *BRAF*^*V600E*^-positive tumors. Also, we chose to compare only patients with documented *BRAF*^*V600E*^ and *RAS*-like mutations, thereby introducing a selection bias. This approach however allows for direct comparison of the clinical effect of these mutations, alone. Given that thyroid molecular testing was not reimbursed by the Quebec’s provincial public medical insurance (Régie de l'assurance maladie du Québec, RAMQ) at the time of data collection, all patients who underwent molecular testing had to cover the cost either via private insurances, at a personal expense or other, which induces a selection bias. Lastly, while the different molecular tests used in this study may have similar diagnostic performances according to the current literature, the lack of uniformity should be considered in the interpretation of these results.

## Conclusion

While USFNA has revolutionized thyroid malignancy diagnostics, the elevated rate of indeterminate results complicates clinical decision-making. In the era of molecular testing, the additional information gained can further inform on tumor behavior. In this study, we found that the *BRAF*^*V600E*^ mutation was associated with more aggressive pathological features than *RAS*-like mutations. Interestingly the mean tumor size of the *BRAF*^*V600E*^ group was smaller. This apparent discrepancy may be explained by the fact that *BRAF*^*V600E*^ positive nodules often show aggressive sonographic features and a higher Bethesda category on USFNA, thereby prompting more diligent work-up. Therefore, according to this study, size in isolation is not as good a surrogate marker for pathological aggressiveness, as previously thought. Mutational status, on the other hand, is a strong predictor of pathological aggressiveness and can thus be useful as a prognostic factor in combination with other clinical, pathological or radiological findings. Further long-term studies are required to solidify the impact of mutational status on other prognostic factors such as rates of recurrence and disease-free survival.

## Supplementary Information

**Additional file 1: Figure S1**. Flow diagram of patient selection process. *NIFTP: Non-invasive follicular thyroid neoplasm with papillary-like nuclear features

**Additional file 2**. Database used for analysis.

## Data Availability

All data generated or analysed during this study are included in this published article [and its supplementary information files].

## Abbreviations

TI-RADS: Thyroid imaging reporting and data system
USFNA: Ultrasound-guided fine-needle aspiration
JGH: Jewish General Hospital
MUHC: McGill University Health Center
ETE: Extra-thyroidal extension
LN +: Lymph node metastasis
MTNS: McGill thyroid nodule score
