# Supplementary material for: Mutational status may supersede tumor size in predicting the presence of aggressive pathologic features in well differentiated thyroid cancer
Source: J Otolaryngol Head Neck Surg. 2022 Mar 4;51:9. doi: 10.1186/s40463-022-00559-9 (PMC8895819; doi:10.1186/s40463-022-00559-9)
Supplement: Supplementary file 1 — Additional file 1: Figure S1. Flow diagram of patient selection process. *NIFTP: Non-invasive follicular thyroid neoplasm with papillary-like nuclear features [file 40463_2022_559_MOESM1_ESM.docx]

**130** Excluded (*NIFTP, benign postoperative pathology, other mutations)

**1651** Total surgeries

**344** Positive pre-operative molecular testing results

**214** Included patients

**97** *RAS-*like mutations

**117** *BRAF^V600E^* mutations

**994** Patients did not undergo molecular testing

**313** No molecular mutations detected

**657** Pre-operative molecular testing
